# A Survey of Practice and Knowledge of Refugee and Migrant Pregnant Mothers Surrounding Neonatal Jaundice on the Thailand–Myanmar Border

**DOI:** 10.1093/tropej/fmw055

**Published:** 2016-08-29

**Authors:** Taco J. Prins, Margreet Trip-Hoving, Moo Kho Paw, Mar Le Ka, Nyo Nyo Win, Gay Htoo, Mu Kaw Hser, Kesinee Chotivanich, François Nosten, Rose McGready

**Affiliations:** 1 Shoklo Malaria Research Unit, Mahidol-Oxford Tropical Medicine Research Unit, Faculty of Tropical Medicine, Mahidol University, Mae Sot, Thailand; 2 Mahidol-Oxford Tropical Medicine Research Unit, Faculty of Tropical Medicine, Mahidol University, Bangkok, Thailand; 3 Centre for Tropical Medicine and Global Health, Nuffield Department of Medicine, University of Oxford, Oxford, UK

**Keywords:** neonatal jaundice, G6PD deficiency, health knowledge, attitudes, practice.

## Abstract

**Background:** In populations with a high prevalence of glucose-6-phosphate dehydrogenase deficiency, practices that can induce haemolysis need to be identified to raise awareness of preventable risks. The aim of this survey was to determine the proportion of prospective mothers using haemolytic agents and their knowledge and practice surrounding neonatal jaundice.

**Methods:** Pregnant mothers were invited to participate in a cross-sectional survey conducted at Shoklo Malaria Research Unit on the Thailand–Myanmar border.

**Results:** From 12 April 2015 to 12 June 2015, 522 pregnant women completed the survey. Mothball use in the household was reported by 41.4% (216 of 522) of prospective mothers and menthol containing products on baby skin by 46.7% (244 of 522).

**Conclusion:** Just over 40% of the households reported use of naphthalene-containing mothballs. Future health promotion activities that focus on reducing naphthalene mothball and menthol-containing products use have the potential to reduce rates of severe neonatal jaundice in this population.

## BACKGROUND

Neonatal jaundice is a common condition affecting 50–60% [1–5] of full-term neonates and 80% [1, 5] of preterm neonates in their first days of their life. Most of the time, serum bilirubin does not reach a high level at which it precipitates in the brain and gives neurological damage, known as acute bilirubin encephalopathy and kernicterus [6]. This is an important problem in middle- and low-income countries in South-Asia and Sub-Saharan Africa, where 73–81 per 100 000 live births develop kernicterus [7, 8], compared with only 0.4–10 per 100 000 live births [2, 6, 7, 9–14] in developed countries. In high-income countries, it is rarely associated with neonatal mortality, in contrast to low- and middle-income countries where it is still an important cause of neonatal mortality [7, 15].

Kernicterus and severe hyperbilirubinemia are strongly associated with glucose-6-phosphate dehydrogenase (G6PD) deficiency [6, 16] and can be exacerbated by haemolytic agents such as menthol and naphthalene [17–20]. Several case reports describe G6PD deficiency and severe hyperbilirubinemia in neonates [21–24], and in two of the cases most likely triggered by the use of naphthalene containing mothballs [22, 23].

Early detection and treatment are the key elements to prevent severe neonatal jaundice. An understanding of the knowledge level in a specific population can help to identify the gaps and target areas for intervention. There is also a need to raise awareness about G6PD deficiency and the danger of the use of haemolytic triggers, because this is a preventable cause of severe neonatal jaundice.

Shoklo Malaria Research Unit (SMRU) provides medical care in rural areas on the border between Thailand and Myanmar, principally for refugees and migrants from Myanmar. The incidence of G6PD deficiency is 7.2–13.7% [25, 26] in this population, and between 2008 and 2011 eight neonates died of kernicterus, four of whom were G6PD deficient [26]. There is no data available on the maternal practice and knowledge surrounding neonatal jaundice in this population and mothballs are available in the area. The primary aim of this survey was to assess mothball use among prospective mothers in the population, as this is amenable to change.

## METHODS

### Ethical approval

This survey was preliminary to a larger study on the aetiology of neonatal jaundice with ethical approval from the Faculty of Tropical Medicine at Mahidol University in Thailand (MUM 2014 032-01). Oxford University (OXTREC 41-14) and the Tak Community Advisory Board (TCAB-4/1/2015) gave ethical approval for the survey.

### Study site and participants

This descriptive cross-sectional survey was carried out in three SMRU clinics: Wang Pa (WPA), Mawker Thai (MKT) and Mae La (MLA) between 12 April 2015 and 12 June 2015. The clinics are located on the north-western border of Thailand and Myanmar, in Tak Province, and patients and healthcare workers are predominantly Karen or Burmese from Myanmar. In these populations, neonates and young infants are traditionally swaddled in baby cloths as described previously [27]. MLA clinic is located in the refugee camp, which is the home for approximately 40 000 refugees [28]. The 2.4 km^2^ total area of the camp implies relative easy clinic access, in contrast to WPA and MKT migrant populations, which are more widely dispersed with several hours of travel required to reach the clinics.

### Study procedures

The knowledge of the pregnant women was obtained through a questionnaire. Only pregnant women attending SMRU antenatal care and who gave written (or thumb print and declaration by a witness) informed consent were invited to participate in the survey.

The questionnaire for pregnant women consisted of 11 questions on neonatal jaundice, divided into four main subjects: recognizing neonatal jaundice, the action she would take if she had a child with neonatal jaundice, the understanding of neonatal jaundice and the use of haemolytic agents (Supplementary file 1). Mothers were tested about the recognition of neonatal jaundice by showing a picture of a neonate with jaundice. In addition, data were collected about the place of residence, parity, age and the time to travel to the clinic. The questionnaires were recorded onto the survey sheets by trained local counsellors, who could speak the local languages, Karen and Burmese, owing to low literacy rate among the population [29].

### Sample size

There is limited data on the prevalence of maternal use of haemolytic agents in this and other regions. One study [30] from Nigeria published in 1985 reported a 45–87% prevalence of the use of haemolytic agents. Using a precision-based sample calculation, 385 women would be required to obtain the true proportion of women who use haemolytic agents with 95% confidence interval and ±10% margin of error if the estimated prevalence was in the order of 50%.

### Statistical analysis

Data were entered in Microsoft Access for Windows 7. Data were exported to Stata 14 (Statacorp, College Station, TX) for descriptive analysis. Fisher’s exact test and the Chi-squared test were used to measure various associations with the knowledge and practice surrounding neonatal jaundice.

The level of significance was set as *p* <  0.05.

## RESULTS

The total number of pregnant women included was 522 of which 283 (54.2%) were from MLA refugee camp and the remainder from the migrant sites: 63 (12.1%) in WPA and 176 (33.7%) in MKT (Table 1). Approximately one in three (37.6%) of the pregnant women were nulliparous. Of note, in the mothers with a previous birth, 61 (18.7%) had a child who had been admitted to special care baby unit (SCBU) because of neonatal jaundice.

**Table 1.** Characteristics of the mothers

Variable	*N* (%) *n* = 522
Surveyed at each clinic, n (%)	
MLA	283 (54.2)
WPA	63 (12.1)
MKT	176 (33.7)
Age, years (mean ± SD), [range]	26.0 ± 6.8 [15-47]
Age groups, n (%)	
<19	101 (19.4)
20–24	154 (29.5)
25–30	127 (24.3)
>30	140 (26.8)
Average travel time to clinic, minutes, mean ± SD [range]	112 ± 362 [1 min–2 days]
Travel time, groups, *n* (%)	
0–59 min (<1 h)	364 (69.7)
60–299 min (1 to < 5 h)	135 (25.7)
≥300 (5 or more hours)	23 (4.4)
Gravida: median (25th, 75th), [range]	2 (1,4) [1-10]
Parity: median (25th, 75th), [range]	1 (0,2) [0-9]
Nulliparous, *n* (%)	196 (37.6)
Previous child admit for neonatal jaundice if Pa ≥ 1, *n* (%)	18.7% (61/326)

Pa  = parity.

Almost half, 216 (41.4%, 95% CI: 37.2–45.6), of the pregnant women reported the use of mothballs. Fewer mothers, 95 (18.2%), reported using them to store their baby cloths and blankets. The use of menthol-containing products on baby skin was high, with 244 (46.7%) of the surveyed mothers reporting use. While the majority of the pregnant women, 483 (92.5%), could recognize jaundice (Table 2) and 498 (95%) thought it was harmful, only 69 (13.2%) provided a reason (liver problem or infection) for why a baby could have jaundice. Healthcare workers were an important information source for neonatal jaundice (Table 2).

**Table 2.** Knowledge and practice surrounding neonatal jaundice of the mothers

	Yes	No	Do not know
	*n* (%)	*n* (%)	*n* (%)
Use mothballs in the house	216 (41.4)	306 (58.6)	NA
Use mothballs to store baby cloths/blankets	95 (18.2)	427 (81.8)	NA
Use menthol-containing cream/ointments on the baby skin	244 (46.7)	278 (53.3)	NA
Recognize jaundice (from photo)	483 (92.5)	39 (7.5)	NA
Identify why jaundice can occur	69 (13.2)	453 (86.8)	NA
Learned about neonatal jaundice from:		NA	NA
Family friends	141 (27.0)		
Midwife/healthcare workers	338 (65.9)		
Traditional birth attendant	1 (0.2)		
Nobody	36 (6.9)		
Action if baby is jaundiced (>1 answer possible)			
Come to the clinic	502 (96.2)	NA	NA
Sun exposure	73 (14.0)		
Herbal/traditional treatment	13 (2.5)		
More breastfeeding	18 (3.5)		
Nothing	2 (0.4)		
Think jaundice can be harmful	498 (95.4)	23 (4.4)	1 (0.2)
Understand consequence of jaundice			
Nothing happens	128 (24.5)	368 (70.5)	26 (5.0)
Baby become sick	500 (95.8)	13 (2.5)	9 (1.7)
Baby become handicapped	435 (83.3)	39 (7.5)	48 (9.2)
Baby can die	446 (85.4)	36 (6.9)	40 (7.7)

The majority of the women, 502 (96.2%), reported they would bring their baby with neonatal jaundice to the clinic and only 13 (2.5%) would use herbal/traditional treatments: eight proposed oral and five topical treatment. Herbal treatment consisted of leaves of different trees that have a special meaning for the Karen, and one mother suggested tobacco leaves.

Knowledge and practice were compared between nulliparous and parous women, and mothers who had a child with neonatal jaundice or not (Table 3). The proportion of women who used sun exposure, traditional treatment and menthol-containing cream or ointment was significantly lower in nulliparous women. A significantly higher proportion of mothers with experience of neonatal jaundice in a former pregnancy were able to provide reasons for the cause of jaundice in comparison to parous mothers without this history (Table 3).

**Table 3.** Knowledge and practice of the mothers by neonatal experience

Variable	Nulliparous (*n* =1 96)	Parous* (*n* = 326)	*p*-value	Parous prior history neonatal jaundice (*n* = 61)	Parous no history neonatal jaundice (*n* = 265)	*p*-value
Recognize jaundice (from photo)	180 (91.8)	303 (92.9)	0.641	58 (95.1)	245 (92.5)	0.589
Identify why jaundice can occur	21 (10.7)	48 (14.7)	0.190	22 (36.1)	26 (9.8)	**<0.001**
Action if baby is jaundiced (>1 answer possible)						
Sun exposure	16 (8.2)	57 (17.5)	**0.003**	18 (29.5)	39 (14.7)	**0.006**
Herbal/traditional treatment	0 (0)	13 (4.0)	**0.003**	3 (4.9)	10 (3.8)	0.716
Think jaundice can be harmful	187 (95.4)	311 (95.4)	0.419	59 (96.7)	252 (95.1)	0.746
Understand consequence of jaundice						
Nothing happens	56 (28.6)	236 (72.4)	0.113	10 (16.4)	62 (23.4)	0.171
Baby become handicapped	159 (81.1)	276 (84.7)	0.298	56 (91.8)	220 (83.0)	0.317
Baby can die	160 (81.6)	286 (87.7)	0.759	57 (93.4)	229 (86.4)	0.590
Use mothballs in the house	89 (45.4)	127 (39.0)	0.147	26 (42.6)	101 (38.1)	0.515
Use mothballs to store baby cloths/blankets	35 (17.9)	60 (18.4)	0.875	13 (21.3)	47 (17.7)	0.516
Use menthol containing cream/ ointments on the baby skin	30 (15.3)	214 (65.6)	**<0.001**	43 (70.5)	171 (64.5)	0.377

Parous: parity ≥ 1. Bold values indicates a *p*-value <0.05.

## DISCUSSION

The reported use of mothballs included 40% (95% CI: 37.2–45.6) of the pregnant population, and one in five mothers reportedly used them to store baby cloths and blankets. Reassuringly, most mothers could recognize jaundice, and if recognized also reported that they would bring their infant to the clinic, reducing delays in diagnosis and treatment [1, 31–33]. Nevertheless, the high use of haemolysing agents in a population with a high rate of G6PD deficiency is undesirable and needs to be reduced.

Our study confers with previous publications reporting health workers as an important information source for neonatal jaundice [31, 34]. Knowledge was higher among mothers with a history of a previous child with neonatal jaundice: most of these cases are likely to have received treatment at SMRU clinics because more than half of the survey participants came from MLA camp where the only neonatal services available are from SMRU. Healthcare workers may not always have a high level of knowledge on neonatal jaundice [32], indicating that regions with a high proportion of G6PD should stimulate learning in this area.

While a higher proportion of parous mothers with a history of a child with neonatal jaundice could say why neonates were jaundiced, this did not result in significantly safer behaviour such as a reduced use of mothballs. The study was not powered to show significance in this respect; nevertheless, it suggests that behaviour change education for all mothers would be useful and differs from previous recommendations to prioritize education of nulliparous women [8, 35].

With the introduction of the SCBU in 2008, SMRU started to treat neonatal jaundice with phototherapy, instead of using sun exposure [26]. The prior use of sun exposure could explain why mostly multiparous mothers still believe that it is a good option. The use of sun exposure has been reported previously from other surveys in South-East Asia [36, 37]. The advantage of using filtered sunlight for the treatment of neonatal jaundice has also been tested [38, 39] with studies showing it is effective, inexpensive and safe, if correctly monitored. One study [40] demonstrated no inferiority of sunlight compared with phototherapy, acknowledging that this was in neonates with bilirubin levels <15 mg/dl.

As sunlight is still reportedly used by some mothers, it is likely that the acceptability of filtered sunlight would be high; however this strategy would not be sufficient to treat severe jaundice.

As naphthalene is insoluble in water, it is difficult to wash it out of the cloths or blankets [20]. A preventive public health initiative to inform the population of the danger to neonates of mothballs used for storage of cloths and blankets, and menthol-containing creams, is essential. This initiative should occur during antenatal clinics and in the community and should also address shop owners of clothes shops. A national ban on naphthalene-containing products, as is the case in the European Union since 2008 [20], would be the ideal scenario for neonates in the future. However, governance and enforcement among marginalized populations are weak. In addition, under humid conditions and crowded settings such as refugee camps there are few alternatives for storage that keep insects from the clothes.

In 2013, the United Nations Refugee Agency relocated approximately 40 000 refugees; approximately 2000 refugees from MLA were relocated all over the world [41]. G6PD deficiency is present in many countries, in some, principally owing to the migration of people, and so, understanding the practice of the mothers and their use of haemolytic agents can be useful to reduce adverse outcomes for the neonate [22]. Basic services on the Thailand–Myanmar border for mothers and babies provided by local trained healthcare staff have significantly improved maternal [42] and neonatal care [26, 43]. As this study suggests that the information on neonatal jaundice comes mainly from healthcare workers, it is important the information that is passed on is correct.

Developing neonatal jaundice educational programme targeting mothers, with an emphasis on avoidance of use of mothballs and menthol-containing creams in the newborn would help in raising awareness and the prevention of neonatal jaundice in the population.

### Limitations

We were only able to survey women attending antenatal care services who may differ from women who do not attend such services. Another possibility is that women in the survey gave an answer that they believed they should do rather than what they do in practice. Nevertheless, the findings are not unexpected and largely consistent with published data.

The strengths of the study include the large sample size, good representation of mother’s ages and detailed questions on practice, which suggest the results are robust enough to advocate for education in the clinic and community.

## CONCLUSION

It is concerning that many expectant mothers in these marginalized populations still report use of mothballs and menthol-containing products, including 18% acknowledging the use of naphthalene to store cloths and blankets for the neonate. While pregnant women are able to recognize neonatal jaundice and have high knowledge of possible complications, most are unware of the causes of neonatal jaundice. Raising awareness of preventable risk factors among prospective mothers and the community, in particular about the risk of haemolytic agents, is an urgent public health priority in populations with a high incidence of G6PD deficiency, with potential to reduce jaundice-related neonatal morbidity and mortality.

## Supplementary data

Supplementary data are available at *Journal of Tropical Pediatrics* online.
